# Radon Survey in Bank Buildings of Campania Region According to the Italian Transposition of Euratom 59/2013

**DOI:** 10.3390/life11060533

**Published:** 2021-06-08

**Authors:** Vittoria D’Avino, Mariagabriella Pugliese, Fabrizio Ambrosino, Mariateresa Bifulco, Marco La Commara, Vincenzo Roca, Carlo Sabbarese, Giuseppe La Verde

**Affiliations:** 1 Department of Physics Ettore Pancini, University of Naples Federico II, 80126 Naples, Italy; vittoria.davino@unina.it (V.D.); laverde@na.infn.it (G.L.V.); 2 National Institute for Nuclear Physics, INFN Section of Naples, 80126 Naples, Italy; marco.lacommara@unina.it (M.L.C.); carlo.sabbarese@unicampania.it (C.S.); 3 Department of Agricultural Sciences, University of Naples Federico II, 80055 Portici, Italy; fabrizio.ambrosino@unicampania.it; 4 Department of Electrical Engineering and Information Technology, University of Naples Federico II, 80125 Naples, Italy; mariateresabiff@hotmail.it; 5 Department of Pharmacy, University of Naples Federico II, 80131 Naples, Italy; 6 Department of Mathematics and Physics of the University of Campania Luigi Vanvitelli, 81100 Caserta, Italy; vroca222@gmail.com

**Keywords:** CR-39 detector, Euratom 59/2013, Italian radiation protection legislation, radon indoor, radon survey

## Abstract

^222^Rn gas represents the major contributor to human health risk from environmental radiological exposure. In confined spaces radon can accumulate to relatively high levels so that mitigation actions are necessary. The Italian legislation on radiation protection has set a reference value for the activity concentration of radon at 300 Bq/m^3^. In this study, measurements of the annual radon concentration of 62 bank buildings spread throughout the Campania region (Southern Italy) were carried out. Using devices based on CR-39 solid-state nuclear track detectors, the ^222^Rn level was assessed in 136 confined spaces (127 at underground floors and 9 at ground floors) frequented by workers and/or the public. The survey parameters considered in the analysis of the results were: floor types, wall cladding materials, number of openings, door/window opening duration for air exchange. Radon levels were found to be between 17 and 680 Bq/m^3^, with an average value of 130 Bq/m^3^ and a standard deviation of 120 Bq/m^3^. About 7% of the results gave a radon activity concentration above 300 Bq/m^3^. The analysis showed that the floor level and air exchange have the most significant influence. This study highlighted the importance of the assessment of indoor radon levels for work environments in particular, to protect the workers and public from radon-induced health effects.

## 1. Introduction

Radon is the heaviest and the only radioactive noble gas present in nature everywhere, generated in rocks and soils throughout the earth’s crust. Its main unstable isotopes, namely ^222^Rn (radon), ^219^Rn (actinon) and ^220^Rn (thoron), are produced in the intermediate steps of the three primordial decay chains of ^238^U, ^235^U and ^232^Th respectively. The radiological importance of each radon isotope depends on its relative abundance and half-life. Due to having the isotopic ratio of ^235^U/^238^U = 0.0072 and a short half-life (T_1/2_ = 3.98 s), ^219^Rn is always ignored. ^222^Rn has the greatest half-life (T_1/2_ = 3.82 d) and has received the most attention from the scientific community in regard to radiation protection, followed by ^220^Rn (T_1/2_ = 56.83 s).

Radon and its progenies are amongst the major sources of the population’s exposure to natural radiation; indeed, they constitute the main contributor to the annual effective radiation among all sources of ionizing radiation [1]. Radon is chemically inert and so does not react with other elements or compounds, and it can easily escape from the ground into the air where it can be inhaled. However, health hazards related to the radon issue are not caused directly by radon, but by its progenies. In fact, because the lifetime of ^222^Rn is longer than the air change time in the human respiratory system, most of the inhaled radon is exhaled and cannot decay with the body. On the contrary, the short-lived radon progeny (^218^Po and ^214^Po) is solid and so reactive that it can attach to atmospheric dust and water droplets forming clusters (attached fraction). Similarly, if inhaled, the decay products of ^222^Rn (unattached fraction) attach themselves to the epithelium of the respiratory system and, due to their short duration, decay. In this way, the alpha particles ionize the DNA structures increasing the probability that, due to the stochastic effect, they can generate carcinogenic processes [2,3].

Since 1988, based on scientific evidences, the International Agency for Research on Cancer (IARC) defined radon as a human carcinogen (group 1) [4] and some decades later the International Commission on Radiation Protection (ICRP) in 2007 [5] and the World Health Organization (WHO) in 2009 [6] identified radon as the second leading causes of lung cancer after cigarette smoking.

Due to its chemical characteristics that allow it to escape easily through rocky substrates and native soils, radon enters buildings through cracks in the foundations or walls and accumulates in indoor environments where it can be breathed by humans.

In addition to the soil, a significant contribution to the accumulation of radon in indoor environments is due to exhalation from building materials of natural origin, in particular with a porous matrix (such as tuff) [7,8].

Furthermore, its indoor concentration is affected by environmental changes such as the frequency of air exchange in a closed environment, and changes outside such as pressure, temperature, and humidity. For this reason, long-term measurements (from 3 to 12 months) that take into account daily and seasonal variations are recommended to evaluate the radon concentration inside a building [9,10]. Thus, the annual average of radon activity concentration provides a representative estimate of indoor radon levels. 

Human exposure to radon occurs both in workplaces and dwellings, since people usually spend a lot of their time in these confined spaces. It has been estimated that people generally spend more than eight hours a day in their workplace; therefore, the monitoring of workers’ exposure is essential [11]. In addition, it is important to assess exposure in confined spaces other than houses (such as schools, shops, offices, banks, hospitals, universities) where significant levels of radon can be observed [12].

Subsequently to the classification of radon among carcinogens, many countries and international organizations have issued norms or recommendations for managing exposure. The WHO recommends the setting of a national reference level as low as reasonably achievable in the range of 100–300 Bq/m^3^ for houses, and the ICRP has also recommended a level not exceeding 300 Bq/m^3^ [5,6]. In Italy, protection against the dangers arising from exposure to ionizing radiation has recently become more prominent with the Legislative Decree 101/2020 [13] which transposed the Basic Safety Standards (BSS) Directive-2013/59/Euratom Directive [14]. Compared to the previous legislation (Legislative Decree 241/2000) [15], the great novelty introduced by the Directive lies in the establishment of protection measurements against the ionizing radiation not only for workers but also for the general population in living environments (Article 19). Furthermore, the Legislative Decree 101/2020 replaced the ‘national action level’ of 500 Bq/m^3^ with the ‘reference level’ of 300 Bq/m^3^ for both workplaces and dwellings (Article 12 comma 1) [13]. The Italian legislation commits employers to evaluate the occupational exposure in fully underground workplaces (e.g., caves, tunnels, cellars, mines, galleries, metro stations, car parks), in thermal structures and in basements and ground floor workplaces of buildings placed in ‘radon-prone areas’ identified and declared by the Regions according to the National Radon Action Plan (Article 10). Remedial actions are required if the annual average activity concentration of radon exceeds the reference level. In the event that the assessment determines a level higher than the reference value, then the employer is asked to calculate the annual effective dose for workers. If the estimation results are lower than 6 mSv, (Article 12 comma 1, letter d) no further actions are required. In this context, the current work presents an extensive measurement survey of radon activity concentration in 62 buildings of a bank company throughout Campania Region, Southern Italy. Very few similar surveys can be found in the literature involving bank buildings spread over all the national territory [16,17]. This Campania region, in accordance with the 2013/59/Euratom Directive and pending for its transposition in the national regulation, approved the Regional Low No. 13/2019 [18] which establishes the reference limit level for the activity concentration of radon gas activity at 300 Bq/m^3^ in all underground rooms, basements, and ground floors of closed environments open to the public, as well as in buildings intended for education and in so-called strategic buildings as declared by the Ministry of Infrastructure [19]. The measurement campaign began in October 2019 and ended in September 2020. The aim of the present study was, firstly, to estimate the annual average radon levels in the underground and ground floors of the banks and then to evaluate remedial actions for these indoor environments where necessary. In order to perform a multifactorial study, data on several factors affecting radon concentration in confined spaces were collected and analyzed. To assess the possible influences and correlations, the results of radon activity concentrations were combined with data on the building characteristics, construction standards, building materials, ventilation conditions and systems, number of doors and windows, and the habits of the occupants.

## 2. Materials and Methods

### 2.1. Study Area and Sampling Design

The banks involved in the measurements survey consisted of 62 buildings, spread across the five provinces of Campania Region: Napoli (44), Salerno (7), Caserta (6), Avellino (3), Benevento (2). 

Campania is a very-interesting area, as its territory is characterized by a large variety of geological environments and a high population density. The geological features, soil characteristics and extensive use of stones of volcanic origin (yellow tuff, green tuff, etc.) in the traditional building construction systems [20] have been considered as responsible for the higher than the national average indoor radon mean activity concentration value (around 70 Bq/m^3^) [21,22,23].

A typical bank building consists of a ground floor where the public are served, in the form of banking halls, offices, conference rooms and office spaces with a daily human occupation of at least eight hours, and often an underground floor arranged in rooms for vaults, deposits, archives and more rarely offices. The building sample object of the study consisted of a total of 136 confined environments, 9 of which were in the underground level.

A CR-39 based detector was placed at each measurement point by the person responsible for safety at work, appropriately trained by our team for correct positioning. Radon measurements were conducted following the recommendations established by the UNI ISO 11665: 2020 standard. A data collection form on building characteristics and occupants’ habits (ventilation system, number of openings, floor and wall cladding materials, number of hours per day of opening doors/windows for air exchange) was requested to be completed.

### 2.2. Radon Activity Concentration Measurement Method

Radon concentration was measured in 136 environments of 62 bank buildings for two consecutive six-month periods. The first period was October 2019–March 2020 and the second period April–September 2020. The mean annual radon activity concentration was calculated as the time-weighted average concentration of the two periods, using the detector exposure times as weights.

Radon activity measurements were performed using Solid-State Nuclear Track Detectors (SSNTDs) of poly-allyl-diglycol-carbonate commercially known as CR-39.

The CR-39 based detector is widely used for integrated and long-term measurement of the radon levels because of its material stability, good ionization sensitivity, stability against various environmental conditions, negligible sensitivity to Thoron, and ease of use [24,25,26,27]. The detection system consists of a closed chamber (Radout®, holder for CR-39 produced by Mi.am srl) through whose walls only ^222^Rn diffuses (not ^220^Rn and ^219^Rn isotope), excluding dust particles and humidity from the measurement volume. During the exposure, the α particles emitted by radon and their daughters interact with the aggregate state of the CR-39 polymer causing damage along its path. The traces of the α particles are then made visible by an optical microscope after a chemical etching of the detector. The etching process consists of immersion of the detector in 25% weight/volume sodium hydroxide (NaOH) solution at 98 °C and 1.181 g cm^−3^ density for 1 h, and then in 2% weight/volume acetic acid (CH_3_COOH) solution for 30 min. Then, the detector is rinsed in distilled water for 1 h in order to stop further etching. The observed track densities were converted into radon activity concentrations using an appropriate calibration factor, which in this case was 0.00209 ± 0.00021 tracks cm^−2^ h^−1^/Bq m^−3^. For the exposure intervals used, we found a detection limit of 4 Bq/m^3^ (obtained with an exposure time of one semester), and our maximum activity concentration resulting of about 700 Bq/m^3^ is far below the saturation limit of the detector [27].

We did not perform thoron measurements in the buildings involved in the study. The device used (CR-39 mounted in a thick wall decay chamber) shows a very low sensibility to thoron (guaranteed by Mi.am srl [28]) so as to obtain radon concentrations that are not significantly affected by thoron interaction. Moreover, for the thoron measurement with CR-39, a different Radout® holder and a different etching process on the detector are required [28,29].

### 2.3. Statistical Analysis

Statistical analysis was carried by verifying the log-normal distribution of radon values using Kolmogorov–Smirnov test. The comparison of radon activity concentration values was performed for the categories ‘ground’ and ‘underground’ level with the non-parametric Mann–Whitney test. Descriptive statistics (median, mean, standard deviation, range, etc.) have been computed on radon annual averages estimated in the two groups. The measurement uncertainty of radon activity concentrations is expressed as expanded uncertainty with coverage factor k = 2 (95% confidence interval). This is a precautionary approach, as indicated in the ISO 11665-3:2020. The metrological relative uncertainty is equal to 14%. Thus, the rooms that showed an annual average radon activity concentration higher than the reference value were classified ‘critical’. Statistical analyses were performed using the Statistical Package for the Social Sciences (IBM SPSS Statistic v.26).

## 3. Results

**Figure 1:**
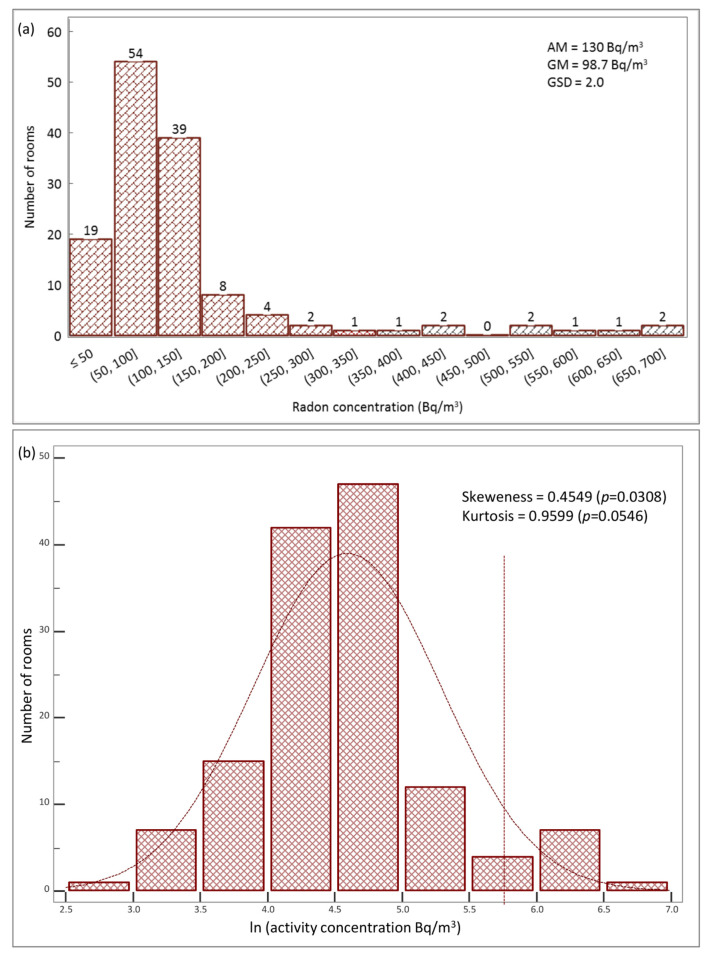
(**a**) Distributions of the annual average radon activity concentration for the full data set (136 bank rooms) expressed as Bq/m^3^. The final bin is an overflow bin that contains all results above 300 Bq/m^3^. Abbreviations: AM, arithmetic mean; GM, geometric mean; GSD, geometric standard deviation; (**b**) Normalized histogram for the natural log of radon measurements fitted with a normal distribution. Vertical dot line indicates the threshold at 300 Bq/m^3^.

Frequency distribution of annual activity concentrations for the 136 rooms is shown in Figure 1a.

Descriptive analysis shows that data distribution is skewed (skewness = 0.45, kurtosis = 0.1), and it is well described by a log-normal model (Figure 1b), checked by the Kolmogorov–Smirnov test (*p* > 0.05, 95% confidence level). In the graph, the values of the geometric and arithmetic means are reported.

**Figure 2:**
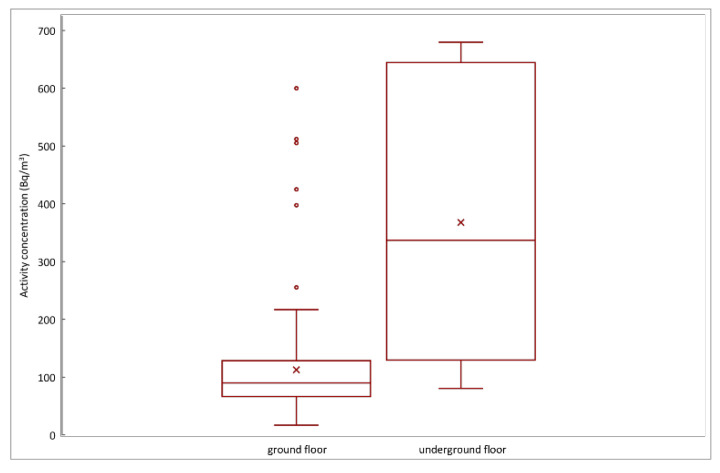
Comparison of annual average radon activity concentration obtained at the ground and underground floors. The graph reports the median, 25th and 75th percentile; the outside values are represented by dots. The cross marker represents the mean value.

Based on the result of the Mann–Whitney test, the significant difference in the annual average radon concentrations between the ground and underground levels was observed (*p* < 0.05) at 95% confidence level. The variation of radon concentration with respect to the different floor level is reported in the box plot of Figure 2.

**Figure 3:**
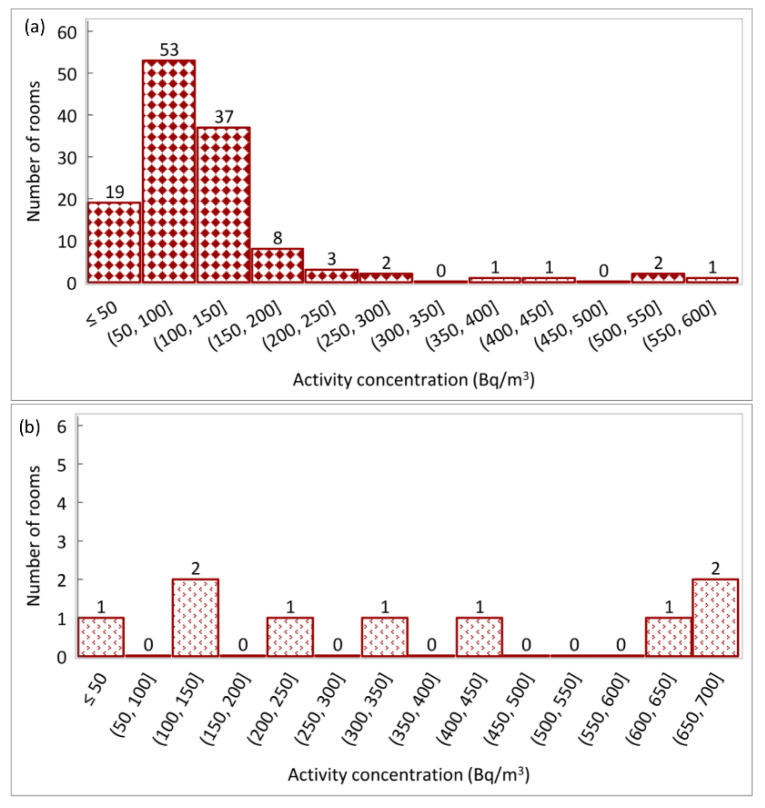
Distributions of the annual average specific concentrations in the (**a**) ground and underground; (**b**) floor levels expressed as Bq/m^3^.

Frequency distributions of the separate annual specific concentrations for the ground and underground floors are reported in Figure 3.

As reported in Figure 3, a total of 10 rooms, five for each category (representing 4% and 56% of the total rooms at the ground and underground levels, respectively), belonging to 7 different buildings, showed a value of the radon concentration exceeding the reference value of 300 Bq/m^3^.

The rooms investigated at the ground and underground floors showed values of the annual average activity concentrations of 17–600 Bq/m^3^ and 80–680 Bq/m^3^, with an arithmetic mean of 113 ± 91 Bq/m^3^ and 368 ± 242 Bq/m^3^, respectively. The median values of 90 Bq/m^3^ and 337 Bq/m^3^ radon concentration were found for the ground and underground levels, respectively. Since the radon results distributions were skewed (Figure 3), the geometric mean was used to describe the central tendency. The results showed geometric means of 91.6 Bq/m^3^ and 286.3 Bq/m^3^ for the ground and underground floors, respectively. A synthesis of the statistic parameters and the number of rooms in which the radon value exceeds the reference level are shown in Table 1.

**Figure 4:**
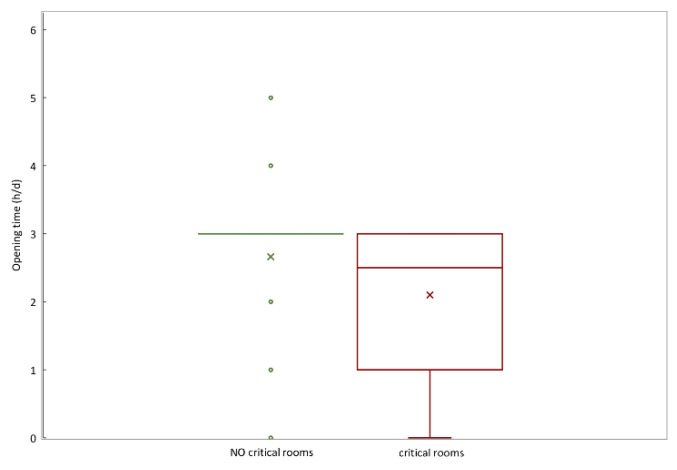
Variability of opening time (h/d) of windows/doors into the groups of ‘critical’ (radon concentration level > 300 Bq/m^3^) and ‘noncritical’ rooms. The graph reports the median, 25th and 75th percentile; the outside values are represented by dots. The cross marker represents the mean value.

The factors affecting radon concentration were investigated. Toward this aim, the rooms with a radon concentration level >300 Bq/m^3^ were categorized as ‘critical’ for the analysis (of all the rooms investigated, 10 were considered critical and the remaining 126 were below the reference value). To verify the existence of any significant difference between the critical rooms and the other ones, the Mann–Whitney test was used. No significant difference in the distribution of the number of openings between the two groups was found, whereas significant change in the variable ‘opening time’ of windows/doors between ‘critical’ and ‘noncritical’ rooms are found (*p* < 0.05) at 95% confidence level. As shown in Figure 4, the range of the mean value of opening time resulted in 2.5–3 h/d in the critical rooms group and 3–5 h/d otherwise. 

The data on the wall cladding materials showed that almost all the analyzed rooms both at the ground and underground levels are plastered (95% and 100% respectively). Similarly, no statistical significance was found with respect to the floor cladding materials for the critical and noncritical rooms of the buildings included in the analysis.

## 4. Discussion

In this study, we analyzed the radon activity concentration in 62 bank buildings spread on Campania region. A total of 136 measurement points (127 at ground floors and 9 at underground floors) were investigated for the annual radon monitoring. Despite that the difference in the sample sizes between the rooms at the underground and ground floors represents a limit for the statistics (it potentially induces a bias), the analyzed sample is the description of the effective distribution of the environments as the monitored buildings belong to a single bank company. The results of the overall data set, expressed in terms of annual average activity concentration, showed a skewed distribution well fitted by a log-normal curve (Figure 1), as expected [30,31]. The distribution of radon activity concentrations is comparable with the results reported in several studies available in the literature [16,21,23,31,32,33,34]. The geometric mean and the geometric standard deviation of the data have been used to describe the distribution, and this knowledge was useful for evaluation of the fraction of rooms that exceeded the reference value (300 Bq/m^3^).

The legislative framework, that was the rationale for this work, plays a key role in the interpretation of the results. Campania Regional Law 13/2019 [18] requires assessment of the radon level in the underground, basement and ground floors of any building with public access, establishing the reference level of 300 Bq/m^3^. According to this law, if the radon activity concentration value exceeds the reference level, the employer must implement remedial actions. Furthermore, compared to the previous Italian Legislative Decree 241/2000, the “reference level” has been introduced replacing the “action limit” and has been reduced from 500 to 300 Bq/m^3^. During these measurements, the transposition of the Euratom 59/2013 directive came into force in Italy that, with respect to the regional law, incorporates all the basic safety standards for protection against the dangers arising from exposure to ionizing radiation. In particular, for radon gas exposure the annual effective dose limit has been increased from 3 to 6 mSv/y, and buildings intended for residential use are involved in the national regulation demanding that regional institutions implement an investment policy to adopt radon reduction strategies, and also for the radioprotection of people at their homes, if required. 

Since the bank buildings include ground and underground confined spaces occupied both by workers and the public, according to Regional Law, a strategy for radon mitigation should be implemented in order to reduce the radon concentration. The owner of the property presents a remediation plan which will be approved by the Municipality (Article 4 comma 3) [18].

The method of choice for mitigation depends on the required reduction factor and the type of floor [35,36]. In general, the best way in which to lower radon levels is to reduce the pressure difference that draws radon into a building [35], but structural interventions are not quick to apply and their feasibility depends on several factors including construction characteristics. One practical method that is immediately applicable is passive ventilation consisting in increasing the number and frequency of opening doors and/or windows, allowing the reduction of indoor radon concentration by dilution (increased volume of fresh air dilutes radon concentration). Many studies in the literature have investigated the impact of passive ventilation through manual airing on indoor radon concentration [37,38,39]. In our study, the significant difference of the hours per day of opening windows and doors between ‘critical’ and ‘noncritical’ rooms supports the potential effectiveness in enhancing the ventilation of the environments. In regard to this, all buildings investigated are equipped both with air conditioners and at least one opening in each room. At the same time, it should be noted that the behaviors of occupants including window opening are influenced by building type, ventilation strategy, heating system, energy characteristics and so on [40]. However, the type of buildings investigated represents a peculiar scenario: inside banks, for security reasons, it is not possible to intervene by increasing the windows opening time, and so it is necessary to design forced ventilation systems that do not alter the degree of building security. Another aspect that plays a fundamental role in managing the risk of radon gas is the intended use of the environments. Our results showed that six rooms are bank archives (five located at the underground floor) exceeding the reference level, with staff access of 18 hours per year, so applying the criteria of Legislative Decree 101/2020 the annual effective dose is lower than the action limit of 6 mSv (Article 12 comma 1 letters c and d). Conversely, two rooms at the ground floor with high radon concentrations are occupied daily by both workers and the public, so according to the criteria of LR 13/2019 the building owner must submit a remediation plan.

From the point of view of the positioning of the rooms in respect to the floor, the statistical analysis found a significant difference between the ground and underground floors (Figure 2). The reason for high levels of radon in cellars could be the contact with soil containing uranium. Many studies in the literature have reported high radon concentration levels in underground sites nearest to the soil and that are usually poorly ventilated (mines, tunnels, underpasses, catacombs, spas, caves) [16,17,23,35,41,42,43,44]. Radon gas enters the building from the ground through cracks, crevices and other leakages or exhales from the walls of the house and, through air flows, spreads and accumulates in the internal environment. The diffusion process and the radon level in a building depends on several factors, such as concentration of radioactivity in the ground, permeability of the ground, nature of the floor and coupling of the building to the ground, ventilation conditions, and lining materials. The highest radon levels occur where each of these factors contribute to increase the radon, but small changes in one or more of them can cause appreciable differences in the radon activity concentration value, even in adjacent buildings of apparently identical construction [45]. This could be the reason for the variability of radon activity concentration found in our data set, ranging from 17 ± 7 to 680 ± 190 Bq/m^3^ with a geometric mean of 98.7 Bq/m^3^ and an arithmetic mean of 130 Bq/m^3^ (Figure 1). In order to reduce the radon concentration to the rooms at ground floor, specific barriers between the cellar and ground floor could help to decrease the amount of radon entering the living areas [41].

Generally, the mean value of annual radon concentration found in the present investigation is higher than the mean national value (77 Bq/m^3^) [30,33,46]. Furthermore, it is interesting to note that the radon values occurring in underground rooms are higher than the mean value reported in the extensive national survey on radon concentration in similar underground workplaces of bank buildings [16]. Conversely, the values of radon activity concentrations found in this study are comparable with other published results deriving from regional campaign of measurements [8,46]. We can speculate that a combination of two factors affects the radon concentration in Campania region: the complex geological and structural setting of this region [47] and the building materials of volcanic rock origin and pyroclastic sediments (i.e., lavic stones, tuffs, pozzolana) presenting high ^226^Ra radioactivity level and used in recent and ancient constructions [8]. It is well known that the radioactivity contents of building materials contribute to radiation exposure, and radon exhalation can increase the radon level indoor, depending on the type of material [7,48,49,50].

In this framework, the knowledge of building materials, construction techniques, occupancy time of the space, combined with a more extensive and homogenous survey involving bank buildings spread all through region could be useful to individuate factors influencing the radon level in the geographical area involved in the survey.

While waiting to enhance the work with future measurement campaigns that could potentially target areas and dwelling types where data are currently sparse, our study provides useful results in the perspective of the imminent implementation of National Radon Action Plan as stated by the Italian Legislative Decree 101/2020 [13]. The plan defines strategies and arrangements for managing exposure to radon in workplaces and homes moving from identification of radon-prone areas (where the radon concentration in a significant number of buildings is expected to exceed the relevant national reference level) by targeted radon measurement survey. Once the radon-prone areas are identified, here the regulation will demand radon measurements in the underground, basement and ground environments both in workplaces and dwellings and, if necessary, the reduction of radon levels within the reference values established for existing and new buildings (Article 12) [13]. In this context, the work focused on the radioprotection issue of workers and the general population in underground and ground environments of buildings opened to the public and where different working activities are performed. 

## 5. Conclusions

This study reports the results of a survey carried out to evaluate the radon concentration in bank buildings in the Campania region of southwestern Italy. The survey covered 62 bank buildings in the five provinces, including 136 closed environments in underground and ground floors. In each room, the radon device was exposed for a period of 12 months. In the underground rooms (such as archives and other rooms not occupied daily by workers) and in poorly ventilated rooms located at ground floors, the average annual radon concentrations were found to be higher than regularly ventilated rooms or those on the ground floor. The difference in radon concentration levels between the two investigated floors confirmed that soil is the main source of indoor radon, and the results also show the effectiveness of increased aeration turnover as a radon reduction strategy. About 93% of the radon activity concentration is below the national reference level of 300 Bq/m^3^. Rooms that exceed the level of 300 Bq/m^3^ (7%) will need remedial actions, such as forced ventilation and specially designed barriers, which could be useful to reduce the radon level. In conclusion, the results highlighted the necessity to increase the radon monitoring in workplaces with a high occupancy factor to ensure the staff and public protection against exposure. Furthermore, the work suggests that the identification of radon-prone areas will provide valuable criteria for implementing targeted radon surveillance and mitigation in workplaces and dwellings in accordance with the Italian radiation protection regulation.

## Figures and Tables

**Table 1:** Statistical data on annual average of indoor radon concentration (Bq/m^3^) in monitored banks by floor level.

Descriptive Statistics	Ground Level	Underground Level
Range (Bq/m^3^)	17–600	80–680
Median (Bq/m^3^)	90	337
AM ± SD (Bq/m^3^)	113 ± 91	368 ± 242
GM (Bq/m^3^)	91.6	286.3
GSD	1.9	2.2
% ^a^ >300 (Bq/m^3^) (No. of rooms)	4 (5)	56 (5)

Abbreviations: AM, arithmetic mean; SD, standard deviation; GM, geometric mean; GSD, geometric standard deviation; ^a^ = percentage of results exceeding 300 Bq/m^3^.

## Data Availability

Not applicable.
